# Temporalis Muscle Transposition in Irreversible Facial Nerve Palsy:‎ A Vestibular Approach

**DOI:** 10.7759/cureus.51348

**Published:** 2023-12-30

**Authors:** Darina Krastinova, Ghaleb A AL-Mekhlafi, Fatma M El-Badawy, Hossein M El-Badawy, Demetrio Germanò

**Affiliations:** 1 Department of Head and Neck Surgery, Cranio-Orbito-Palpebral Surgery Unit, Hôpital Foch, Suresnes, FRA; 2 Department of Medicine, Fakeeh College for Medical Science, Jeddah, SAU; 3 Department of Oral and Maxillofacial Radiology, Faculty of Dentistry, Ain Shams University, Cairo, EGY; 4 Department of Pharmacology and Toxicology, Taibah University, Madinah, SAU; 5 Department of Maxillofacial Surgery‎, Ospedale dell’Angelo‎, Venice, ITA

**Keywords:** zygomatic arch, cranial nerve vii palsy, parotid tumour, vestibular approach, unilateral facial nerve palsy

## Abstract

Background

The facial nerve plays a crucial role in innervating the motor supply of facial muscles, enabling essential facial expressions that facilitate human communication. Defects or damages to this nerve can have significant consequences, leading to functional, emotional, and social difficulties caused by the immobility of facial muscles. Patients suffering from irreversible facial nerve palsy often experience functional symptoms such as eyelid closure defects, mouth deviation, and limited movement.

Methods

This study aims to address these challenges and offer potential solutions for patients with irreversible facial nerve palsy. In this study, 18 patients (three males and 15 females) underwent temporalis transfer with an intraoral approach in the cranio-orbito-palpebral unity. Preoperative facial reanimation planning involved evaluating the smile's appearance on the unaffected side. Photographs were captured in various positions, and facial expressions were examined. Following this, botulinum toxin was injected into the normal side of the face seven days before the procedure to address the hyperactive condition of the mimic muscles.

Results

Temporalis transfer with an intraoral approach for oral commissure reanimation in the context of irreversible facial nerve palsy was performed. The surgical procedure combined coronal and orbital approaches and achieved the desired outcomes. Postoperative complications observed included hematomas and ossification. Functional outcomes, assessed using the House-Brackmann grading system, indicated a mean improvement of 2.5, signifying moderate dysfunction with normal tone and facial symmetry.

Conclusion

This intervention represents an alternative approach to actual techniques of facial palsy reanimation, especially in selected patients who can benefit from the absence of visible scars, such as young patients and those prone to hypertrophic and keloid scarring, as well as patients with non-prominent nasolabial folds.

## Introduction

The facial nerve is of utmost importance as it provides motor innervation to the facial muscles, which is important for all facial functions and allows for essential facial expressions that play a significant role in human communication. Over time, various treatments have been developed to address the effects of facial nerve palsy successfully. In surgical practice, the first stage involves addressing the oral commissure, followed by planning for eyelid reconstruction. A reliable dynamic technique used to reanimate the oral muscles in cases of irreversible facial palsy is the transposition of the temporalis muscle [1]. However, this technique has traditionally led to external scars and damage to the temporal region, affecting aesthetics.

The facial nerve, also known as the seventh cranial nerve (VII), is a large motor nerve originating from the midbrain, precisely from the pons. The facial nerve courses through the subarachnoid space to reach the internal auditory meatus within the petrous part of the temporal bone, where it emerges at the styloid foramen. Continuing its course, it sustains into the parotid gland to supply 16 muscles responsible for facial expressions on each side [2].

The facial nerve has three terminal branches: The first one supplies the orbicularis oris muscle that mainly controls the movement of the mouth; the second branch supplies the orbicularis oculi that is responsible for the closure of the eye; and the final branch called the frontal branch has multiple branches that mainly traverse the temporal region to innervate the frontalis, corrugator, procerus, and depressor supercilii muscles [3]. The temporalis muscle lies within the temporal fossa, originating from the inferior temporal line and inserting at the apex and medial side of the coronoid process of the mandible. The temporalis muscle is divided into three parts, each supplied with independent vascular supply and innervation. The anterior and middle third are vascularized by the deep temporal artery and a branch of the maxillary artery [4]. The anterior part constitutes about 30% of the muscle mass and is supplied by the anterior deep temporal artery, while the central part forms approximately 51% of the muscle mass and is supplied by the posterior deep temporal artery. The posterior third of the temporalis muscle forms only 19% of the muscle mass and receives its blood supply from the medial temporal artery and a branch from the superficial temporal artery [5,6]. In general, the main blood supplies are in close proximity to the innervating branches. The temporalis muscle is primarily innervated by the deep temporal nerves, which are branches of the mandibular nerve (V3), a division of the trigeminal nerve. The deep temporal nerves provide motor innervation to the temporalis muscle, allowing for its contraction and involvement in activities such as chewing and biting. The deep temporal nerves divide into anterior and posterior branches to supply the corresponding portions of the temporalis muscle. The anterior branch innervates the anterior part of the muscle, while the posterior branch innervates the central and posterior portions.

The impact of facial nerve palsy extends beyond physical impairments, significantly affecting the quality of life. In addition to the inability to close the eyelids adequately, oral asymmetry can lead to speech difficulties and psychological manifestations. Both the impairment of muscle movements and irreversible facial nerve palsy contribute to functional deficits related to mouth and eyelid movements.

## Materials and methods

Study participants

Eighteen patients, comprising three males and 15 females, underwent temporalis transfer with an intraoral approach [7] in the cranio-orbito-palpebral unity. The participants' ages ranged from 27 to 60 years, with a mean age of 44 years. All individuals presented with irreversible facial nerve palsy that had persisted for over 18 months.

Ethical approval

Ethical approval for this study was obtained from the Ospedale dell'Angelo ethical committee, ensuring that the research adhered to all ethical standards and regulations. Confidentiality was rigorously maintained throughout the study. No data that could potentially reveal the patients' identity, such as names or personal information, were utilized. Consents were obtained for medical photography, and images were partially covered to ensure the anonymity of patients.

Surgical procedure

All patients received oral commissure reanimation through a temporalis transfer with an intraoral approach. Approximately 66% of the participants underwent postoperative education for a minimum of 12 months, with a mean duration of 13.5 months, to facilitate their postoperative recovery and optimize outcomes.

Outcome assessment

To evaluate the surgical outcomes comprehensively, a combination of clinical examinations and the House-Brackmann grading system was employed both before and after the interventions. The House-Brackmann grading system, a widely recognized tool for assessing facial nerve function, was utilized to quantify the severity of facial nerve palsy and track improvements post-surgery [7,8].

## Results

Preoperative period

The cause of palsy was found to be traumatic in six patients, an VIII nerve tumor in three patients, and a parotid tumor in nine patients. Table 1 shows the causes of facial nerve palsy.

**Table 1:** Causes of facial palsy

Cause of palsy	Patients
Post-traumatic	6
VIII nerve tumor	3
Parotid tumor	9
Total	18

**Figure 1:**
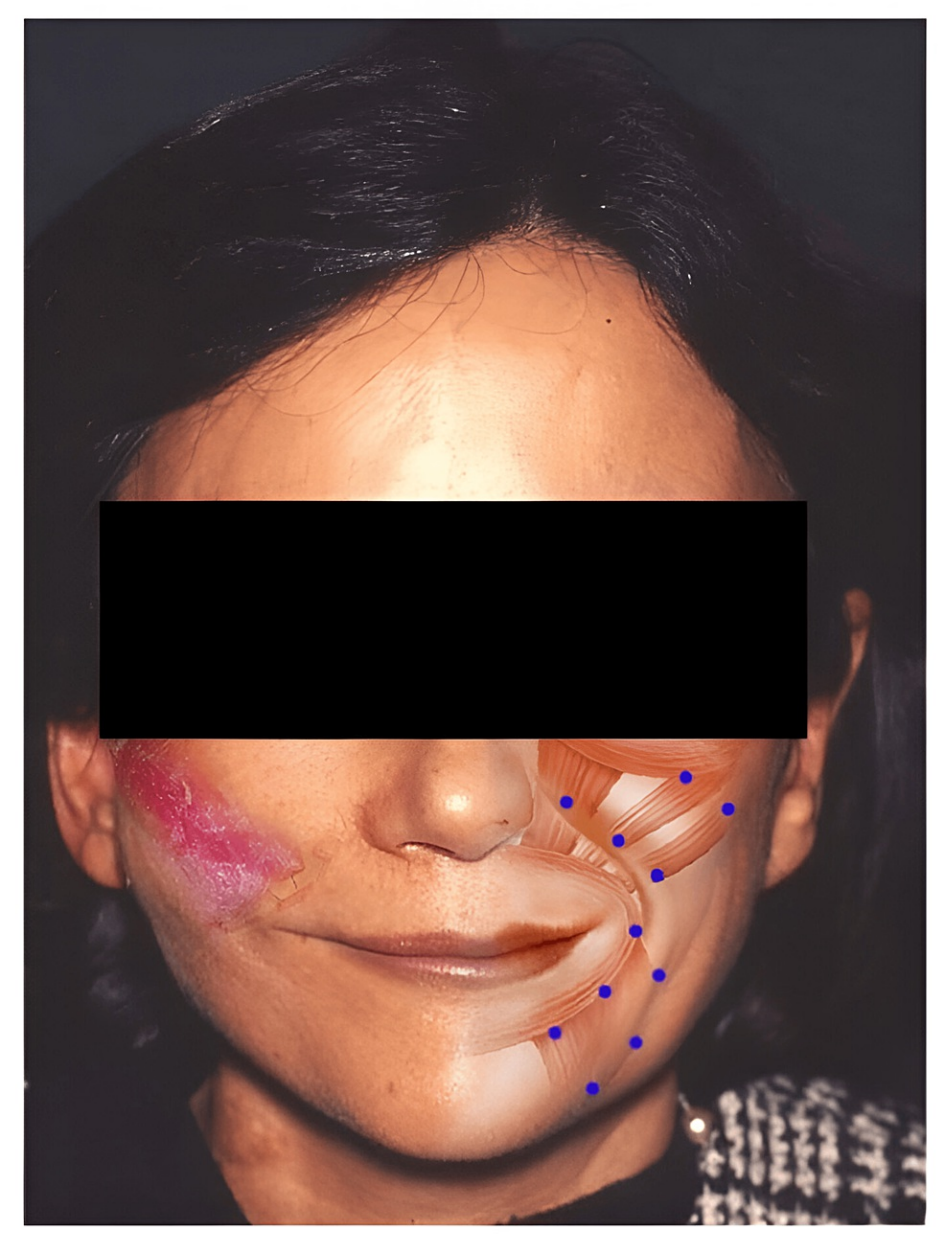
Injection points of botulinic toxin on the normal side This figure shows the sites of botulinic toxin injection administered to the unaffected side of the face seven days before surgery.

The planning of preoperative facial reanimation in this study consisted of assessing the appearance of the smile on the normal side. Additionally, photographs were taken in different positions, and facial expressions were analyzed. Subsequently, the normal side of the face was injected with botulinum toxin seven days before the procedure to correct the hyperfunctioning state of the mimic muscles (Figure 1).

Surgical technique and description of procedures

**Figure 2:**
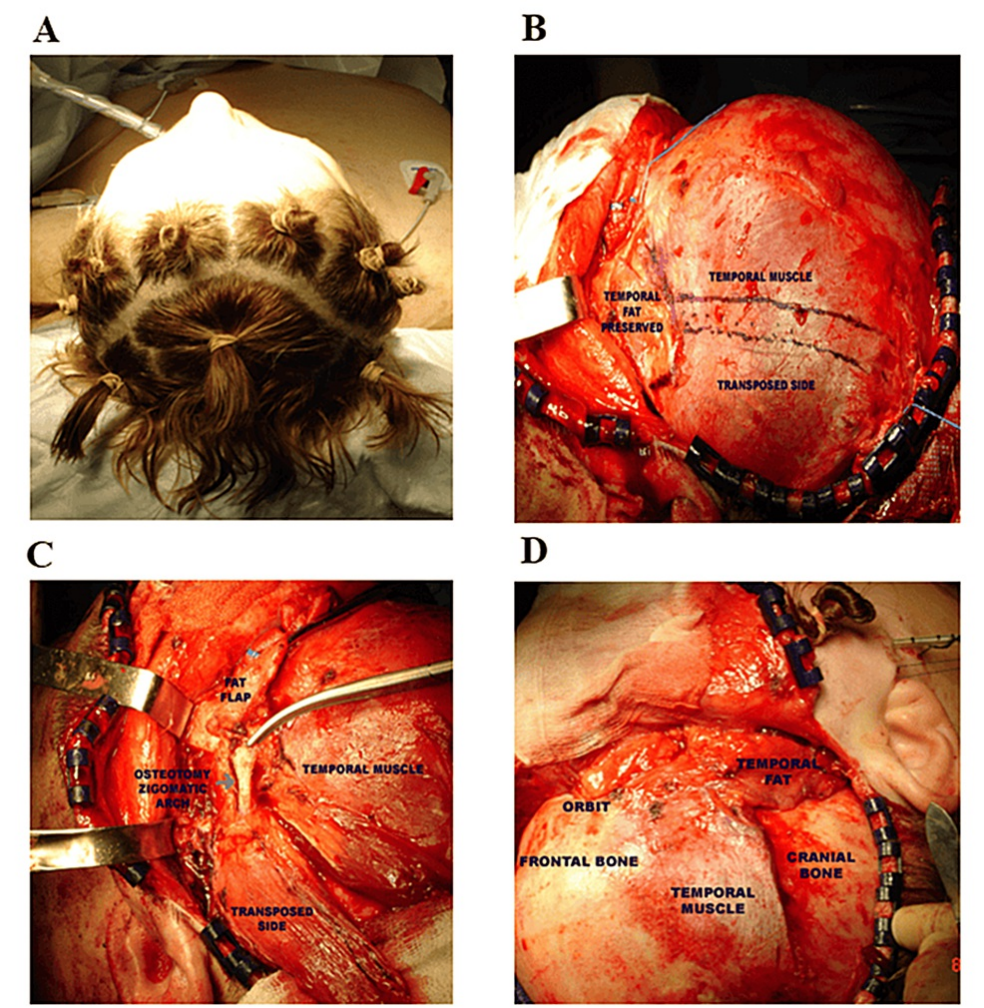
Surgical technique and procedure Preoperative mark of the coronal approach (A), dissection over the temporalis fascia and protection of temporal fat pad (B), osteotomy of the zygomatic arch (C), and transposition of the temporalis muscle (D).

A coronal approach was used with an incision made along the natural hairline on the scalp. This hairline incision cut minimizes the visibility of scars as it follows the natural contour of hair growth, making it less noticeable. The coronal approach involves making an incision along the coronal suture of the skull, which is the line where the frontal and parietal bones of the skull meet. This incision typically runs from one ear to the other, passing over the top of the head. Afterwards, a subperiosteal dissection was performed as previously reported [9]. The dissection extended to the temporal region, and then the periosteum was elevated as shown in Figure 2. Frontal dissection was carried out underneath the periosteum at the subperiosteal plane, extending in the direction of the supraorbital rims, up to 2 cm above the supraorbital rim.

To release supraorbital nerves, the orbital approach was used. The orbital approach preserves the integrity of the orbital structures, while the dissection was carried out in the subperiosteal layer, where the nerve pathway is located, making it non-traumatic. The dissection of the subperiosteal layer proceeded from the supraorbital rim to the lateral orbital wall and lateral orbital rim. Upon completing the dissection of the inferolateral orbit, the upper part of the zygomatic arch was exposed along with the curve of the deep temporal fascia. After dissecting the subperiosteal layer of the lateral orbital rim at the zygoma plane, they are joined using a periosteal elevator. The final stage of the orbital dissection is carried from the zygoma to the malar prominence.

Osteotomy of the zygomatic arch was subsequently performed. The deep temporalis fascia was incised in the middle of the muscle, and the subperiosteal dissection was continued. A portion of the posterior half of the temporalis muscle, along with the posterior two-thirds of the overlying fascia, was elevated. To protect the temporal fat pad, it was carefully preserved and fixed over the anterior part of the temporalis muscle, which remains in its original position. Preserving the temporal fat pad is important in providing volume in the subcutaneous plane, partially filling the void left by the transposed muscle.

Next, needles were inserted on the cutaneous side of the nasolabial fold to illustrate the intraoral projection, where the incision of the mucosa will be carried out. Thereafter, a deep dissection was performed to create a subcutaneous tunnel up to the superficial muscular aponeurotic system (SMAS) layer, extending in the direction of the zygomatic arch. Another tunnel was created in the zygomatic region on the same surgical plane, and the deep tunnel was then connected carefully with minimal disruption.

Then, a 2.0 non-absorbable suture was anchored at the lower end of the muscle flap, and a Kelly clamp was inserted through the tunnel, from the buccal mucosa to the zygomatic region, to grasp the suture. Subsequently, the temporal flap was pulled toward the buccal region, and the muscle was secured in the angulo-mandibular region by suturing. This allows for the stabilization of the newly transposed flap and prevents retraction from the oral side. Additionally, the temporal fascia was fixed at the oral commissure as per the preoperative plan.

**Figure 3:**
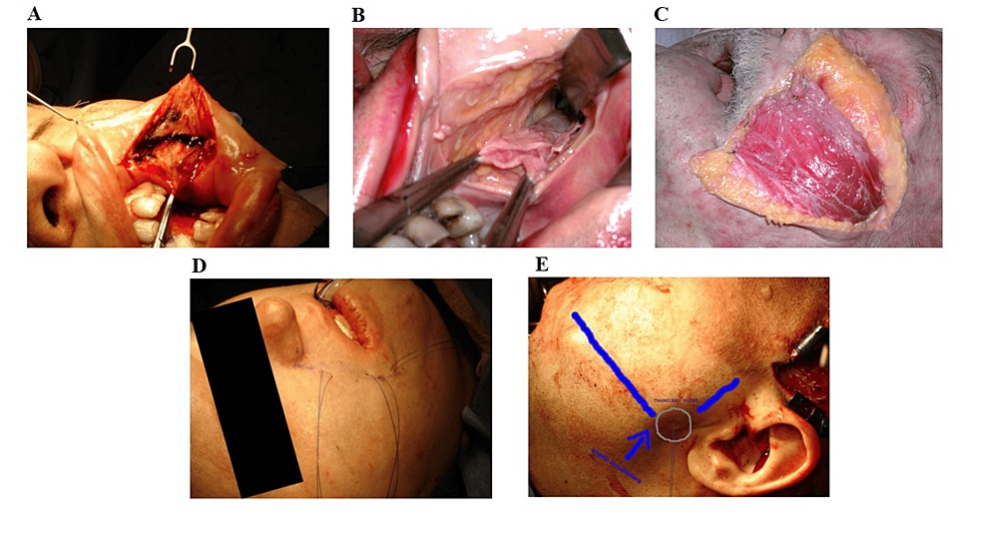
Osteotomy of the zygomatic arch‎ The intraoral approach (A), the up SMAS plane of dissection shown on a cadaver (B), the transposition of temporalis flap (C) shown in a cadaver, the muscle fixation in the nasolabial fold (D), and the fixation of the flap in the angulo-mandibular region (E). SMAS: Superficial musculoaponeurotic system.

Later on, a lateral canthopexy and SMAS fixation were performed on the deep temporal fascia of the anterior temporalis region, using 2.0 silk sutures. Afterward, the zygomatic arch was placed in the temporalis fossa, just behind the anterior part of the temporalis muscle, and a polyethylene terephthalate (PET) patch was inserted into the anterior temporalis fossa. Subsequently, the intraoral and scalp incisions were sutured, and drains were bilaterally placed in the region of the temporal dissection, followed by the application of a suitable dressing (Figures 2, 3).

Several complications occurred postoperatively with certain approaches, such as in the coronal approach that resulted in forehead and scalp hematoma. Additionally, a complication related to the intraoral approach led to hematoma formation in the temporal flap and lip edema. Finally, an atypical complication of our intervention was ossification of the superficial side of the transposed flap.

Postsurgical results

**Figure 4:**
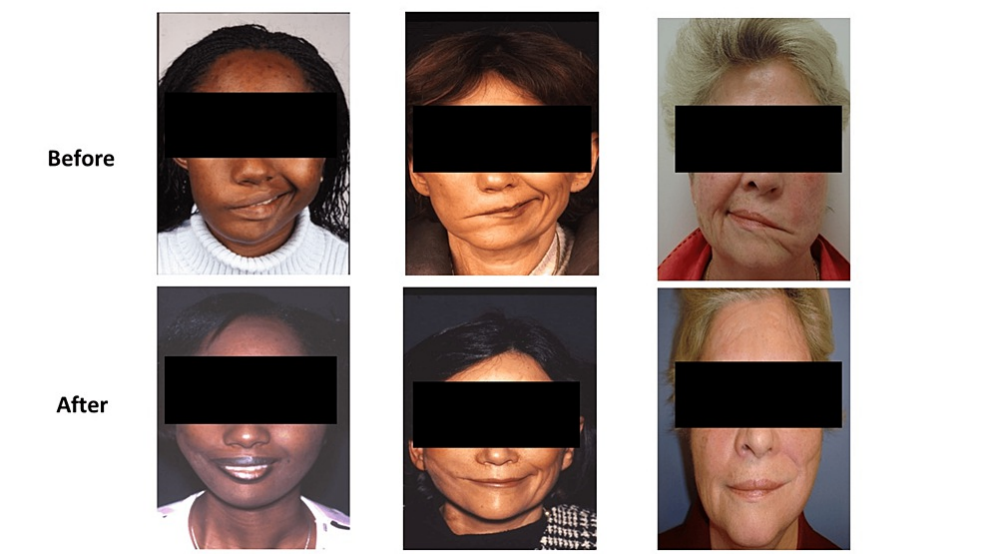
Cases before and after surgery This figure illustrates the sample of cases before and after surgery, showing improvement results.

In this study, 18 candidates underwent temporalis muscle transposition, and there were no occurrences of intraoperative complications. Regarding postoperative complications, three participants developed intra-temporalis hematomas, and one experienced flap necrosis within early periods, necessitating surgical exploration. Additionally, in the late postoperative periods, one participant presented with ossification of the newly transposed temporalis facia. Functional outcomes of our participants were identified using the House-Brackmann grading system, with the mean point of 2.5 indicating moderate dysfunction. In addition, the tone and symmetry of face were normal (Figure 4), despite that global loss of mobility was noted. The mean improvement of the House-Brackmann grading was 2.5. The functional outcomes are shown in Table 2.

**Table 2:** Functional results This table depicts the functional results showing House-Brackmann grading (H.B. grading) before and after surgery.

H-B grading	Before surgery (patients)	After surgery (patients)
I	0	0
II	0	3
III	0	6
IV	0	9
V	3	0
VI	15	0
Total	18	18

Indications

Our technique is particularly indicated for young patients and those with a predisposition to hypertrophic and keloid scar formation, such as individuals of African descent. The rationale behind this approach is to avoid external scarring throughout their lifetime and to prevent the occurrence of hypertrophic and keloid scars. Another indication for this technique is in patients with non-prominent nasolabial folds.

## Discussion

Patients with facial paralysis require reanimation procedures that involve various surgical techniques [10-12]. Moreover, myoplasties are surgical techniques applied to the muscles with different innervation patterns, resulting in the restoration of normal functions performed by the paralyzed mimic muscles. Furthermore, anatomo-physiological data advances the choice of a temporalis myoplasty [12-14]. Additionally, reanimation of a paralyzed face requires the action synchromism of mimic muscles, the nearness, and the presence of the trigeminal facial reflex [15]. Our technique has utilized the posterior half part of the temporalis muscle as a rotatory flap, with an internal approach on the oral side, to restore the oral commissure in irreversible facial palsy. Additionally, the utilization of this technique showed anatomical, functional, and aesthetic advantages. The anatomical advantage of this technique showed that the flap has a reliable vascular supply and innervation pattern.

Vascularization relies on the anterior deep temporal artery, the medial temporal artery, a branch of the superficial temporal artery, and branches of the maxillary artery. As for the innervation of the flap, it is provided by two branches of the mandibular nerve, the deep temporal nerves, and the motor branch of the masseteric nerve [16].

Regarding the functional advantages, pulling the transposed temporalis muscle to the oral sphincter without significant damage to the masticator function is presented due to the preservation of the anterior mid-portion of the temporal muscle. Furthermore, the transposition of the temporalis flap provided dynamic rehabilitation of the oral commissure and jugal region, additionally pulling up the inferior eyelid dynamically.

The House-Brackmann grading represents the quantification values of the functional results, showing that our patients progressed from complete facial palsy to moderate dysfunction [17]. The mean improvement of the House-Brackmann grading was 2.5.

As for the aesthetic advantages of our technique, it provided an absence of scars and preservation of temporal prominence. Scars were not visible due to the combination of the coronal with the internal approach on the oral side. Furthermore, temporal depression was avoided by protecting the temporal fat pad, replacing the zygomatic arch, and preserving the anterior half part of the temporalis muscle. However, this technique had a high rate of temporal flap hematoma formation. Hematomas on the oral side are more common than those on the extraoral approaches. The therapeutic protocol we used after the temporalis transposition was to provide ultimate surgical modification, continuation of the treatment with botulinic toxin on the normal side (Figure 1), postoperative re-education on the affected side, and adjustment of surgical treatment of the paralyzed eyelids.

The main challenges in patients with irreversible facial palsy were not only functional and aesthetic but also psychological due to the difficulty in having the same quality of life as before the onset of the procedure. Our intervention resolves functional and aesthetic problems, but it appears that patients did not completely overcome the psychological problems. On the other hand, the improvement of the mimic functions given by the surgical intervention can assist the patients in beginning postoperative re-education, leading to engaging in social activities [18]. The confirmation of this is that all patients were satisfied with the surgical intervention, and there were no regrets about undergoing the operation. While facial rehabilitation may not achieve a flawless restoration of the original appearance and function before the onset of a condition, targeted intervention can significantly enhance facial muscle motor control. The overarching objective is to improve physical function, enhance appearance, and boost patient self-confidence. Striving for realistic functional outcomes remains a key aspect of this approach [19].

Temporalis muscle transposition was previously assessed in facial paralysis patients with a 26-month average follow-up period. About 80% of patients demonstrated positive outcomes [20]. With a follow-up of up to one year, with a modified orthodromic temporalis muscle transfer technique without zygomatic arch resection, a study showed that immediate results are maintained for one year at least [21]. Comparing long-term and short-term postoperative outcomes, restored facial functions showed a significant improvement in the long term, indicating that satisfactory House-Brackmann score immediately after surgery is most probably going to improve over time [22]. For enduring facial palsy, the prevailing method for restoring smile functionality is the gracilis-free functional muscle transfer (FFMT), acknowledged as the benchmark approach. An alternative to employing muscle-free flaps is the utilization of local muscle transposition, with a particular emphasis on temporalis muscle transposition as the favored procedure. There has been a resurgence of interest in pedicled regional muscle flaps, including the temporalis muscle flap. Of note, the gracilis flap is typically revascularized, allowing for a reliable blood supply, while the temporalis muscle flap relies on its native blood supply, which involves the deep temporal arteries. It is a pedicled flap, meaning it maintains its original blood supply. The gracilis flap can be innervated by the contralateral facial nerve, masseteric nerve, or both, while the temporalis muscle flap retains its nerve connections as it is innervated by the deep temporal nerve. Comparative analysis between these two techniques revealed no notable disparities in the postoperative quality of life or levels of depression/anxiety. Opting for gracilis FFMT does not yield superior results in terms of both quality of life and aesthetic outcomes when compared to temporalis muscle transposition [23].

The study exhibits, however, few notable limitations. The relatively small sample size of 18 patients may restrict the generalizability of the findings to a broader population. Being a single-center study, the results may not fully represent the diversity of outcomes seen in different healthcare settings. Also, the short follow-up durations may not capture long-term outcomes and complications comprehensively.

Heterogeneity in the etiology of facial nerve palsy and variability in surgical expertise among different surgeons could introduce confounding factors. Recognizing these limitations is crucial for a comprehensive understanding of the study's scope and findings. Furthermore, suggesting directions for future research to address these limitations and further investigate the procedure's outcomes can enhance the study's contribution to the field.

## Conclusions

In this study, temporalis muscle transfer was successfully performed via an intraoral approach in patients with irreversible facial nerve palsy, all presenting with symptom durations under 18 months. The combination of coronal and orbital approaches yielded optimal surgical results. Postoperative complications included hematoma and ossification. Notably, the House-Brackmann grading system showed a mean postoperative score of 2.5, indicating moderate dysfunction but with normal tone and facial symmetry, and a significant 2.5-point improvement. This technique is particularly relevant for young patients and those prone to hypertrophic and keloid scars, offering a method to minimize external scarring and related risks, especially in individuals with non-prominent nasolabial folds.
